# Prevalence and Risk Factors for Latent Tuberculosis Infection among Health Care Workers in China: A Cross-Sectional Study

**DOI:** 10.1371/journal.pone.0066412

**Published:** 2013-06-18

**Authors:** Xia Zhang, Hongyan Jia, Fei Liu, Liping Pan, Aiying Xing, Shuxiang Gu, Boping Du, Qi Sun, Rongrong Wei, Zongde Zhang

**Affiliations:** Beijing Chest Hospital, Capital Medical University, Beijing Key Laboratory of Drug Resistance Tuberculosis Research, Beijing Tuberculosis and Thoracic Tumor Research Institute, Beijing, China; St. Petersburg Pasteur Institute, Russian Federation

## Abstract

**Background:**

Health care workers (HCWs) are at risk of latent tuberculosis infection (LTBI). In China, tuberculosis (TB) is a major public health problem, but the prevalence of LTBI in HCWs especially in the hospital for pulmonary diseases has not been assessed enough. The aim of this study was to determine the prevalence and putative risk factors of LTBI among HCWs in a chest hospital and a TB research institute in China.

**Methodology/Principal Findings:**

A cross-sectional study was conducted among HCWs in China in 2012. LTBI was assessed by T-SPOT.TB, and information on HCWs was collected using a standardised questionnaire. Risk factors for LTBI were analyzed by univariate and multivariate regression. The overall prevalence of LTBI among HCWs was 33.6%. Analyzed by job category, the highest prevalence was found among laboratory staff (43.4%). In the different workplaces, the proportion of LTBI was significantly higher among the high risk workplaces (37.4%) compared to the low risk workplaces. The duration of employment had a significant impact on the prevalence of LTBI. Positive T-SPOT.TB test results accounted for 17.6%, 16.8%, 23.5%, 41.8% and 41.6% in groups of ≤2, 3–5, 6–10, 11–20, and >20 working years respectively. In multivariate analysis, job categories (Laboratory staff [2.76 (95% CI: 1.36; 5.60)], technician staff [2.02 (95% CI: 1.12; 3.64)]); working duration as a HCW for 11 to 20 years [3.57 (95% CI: 1.46; 8.71)], and 20 years above [3.41 (95% CI: 1.28; 9.11)]; and the history of household TB contact [2.47 (95% CI: 1.15; 5.33)] were associated with increased risk of LTBI.

**Conclusions/Significance:**

Prevalence of LTBI estimated by T-SPOT.TB is high among Chinese HCWs and working duration, job category and the history of household TB contact were associated with increased risk. These data highlight adequate infection control measures should be undertaken.

## Introduction

China has the world’s second largest tuberculosis (TB) epidemic, behind only India, with more than 1.3 million new cases of TB every year [1]. The high incidence of multidrug-resistant (MDR) TB in China is an issue that poses a challenge to infection control measures [2]–[4]. The higher number of patients seeking treatment at health facilities increases the exposure of health care workers (HCWs) to TB. Current evidences indicate an increased risk of TB among certain groups of HCWs compared to the general population [5]–[7], and this has been heightened by the emergence of multidrug resistant strains of *Mycobacterium tuberculosis* (MTB) [8].

According to a systematic review, in low and middle income countries, the prevalence of latent tuberculosis infection (LTBI) in HCWs ranged from 33% to 79%, with a pooled prevalence estimate of 54%. The median annual incidence of TB infection in low and middle income countries attributable to HCWs has been estimated at 5.8% [8]. Only one study has documented the prevalence of LTBI in HCWs in China. It evaluated the prevalence of LTBI in HCWs in TB centers in Henan province using tuberculin skin testing (TST), and showed that the LTBI prevalence of HCWs with and without Bacillus Calmette-Guérin (BCG) scar was 55.6% and 49.0% [9]. However, the specificity of TST is limited due to PPD (BCG-vaccination strain) cross reactivity and several non-tuberculous mycobacteria (NTM) [10]. These limitations of TST were overcome by a newly developed diagnostic test designated as *in-vitro* interferon-gamma release assay (IGRA).

IGRA is highly MTB-specific and based on the measurement of gamma-interferon (IFN-γ) production from peripheral blood mononuclear cells in response to two MTB secreted proteins, early secreted antigenic target six protein (ESAT-6) and culture filtrate protein 10 (CFP-10), which are absent in the vaccine strain BCG thus not confounded in populations containing a high proportion of BCG-vaccinated individuals [11], [12]. Two commercial systems (QuantiFERON–TB Gold, Cellestis, and T-SPOT.TB, Oxford Immunotec) have been used and reported that LTBI could be detected more specifically than TST [13], [14]. Recent guidelines recommend that these tests be used instead of TST [15].

As there are limited data on the epidemiology and risk of LTBI in HCWs especially in the hospital for pulmonary diseases and there is no published study describing LTBI by IGRA in China, the aim of this project was to evaluate the prevalence of LTBI and putative risk factors of LTBI among HCWs in a chest hospital and a TB research institute in China.

## Methods

### Ethics Statement

A written informed consent was obtained from each participant. The study was approved by ethical committees of Beijing chest hospital.

### Study Design and Setting

We conducted a cross-sectional study from June to August 2012 at Beijing Chest Hospital, Capital Medical University and Beijing Tuberculosis and Thoracic Tumor Research Institute in China. The hospital is a specialist tuberculosis and thoracic tumor disease clinic, with a total capacity of 533 beds. It includes three tuberculosis wards, three thoracic tumor wards, three thoracic surgery wards, one bone tuberculosis ward, one critical care unit, one out-patient service, three radiology departments, one clinical chemistry laboratory, one pathology department, and one administrative department. The tuberculosis research institute includes TB laboratories and non-TB laboratories.

### Participants and Data Collection

Of 828 HCWs in the hospital and the institute, 787 (95.0%) answered the questionnaire and agreed to be tested for LTBI. Sample size calculation was based on a prevalence rate of 40% in a previous survey in a high TB burden country [16]. At α error of 0.05 and β error of 0.20 and 15% non response rate, the minimum sample size required was 166. Individuals with positive T.SPOT.TB without symptoms and computed tomography (CT) findings compatible with active TB were considered as carriers of LTBI. Individuals with CT findings compatible with active TB and a history of TB were excluded from the study. Each individual enrolled into the study completed a standardized questionnaire: age, gender, education, job category, workplace, number of years employed as a HCW, the history of household TB contact, BCG vaccination. BCG vaccination was assessed through the vaccination register. If no register is available, vaccination status is verified by scars. Work areas with routine TB patients or specimens contact were defined as high risk workplaces, whilst those with no routine TB patients or specimens contact were classified as low risk workplaces. For all individuals, the blood was collected for IGRA. HCWs identified as LTBI positive within the medical service were offered the potential value of IGRA and counseling on the risks and benefits of chemoprophylaxis.

### T-SPOT.TB Test

The T-SPOT.TB test, (Oxford Immunotec, Oxford, UK), was used according to previously described [17]. Briefly, eight millilitres of blood was drawn from each subject by venopuncture in a vacutainer CPT tube (Beckton Dickinson Diagnostics, Franklin Lakes, NJ). Peripheral blood mononuclear cells (PBMCs) were isolated by centrifugation, washed twice, and were re-suspended in GIBCO AIM-V (Invitrogen, Auckland, N.Z). PBMCs at a concentration of 250,000 cells/well in AIM-V cell culture medium (Invitrogen Corporation, Carlsbad, USA) were stimulated with ESAT-6 and CFP-10 in 96-well plates pre-coated with anti-IFN-γ capture antibodies, and incubated overnight at 37°C in 5% CO2. After the incubation, wells were washed four times with PBS and incubated for one hour at 2–8°C with a monoclonal antibody to IFN-γ conjugated with alkaline-phosphatase. After another four washing steps and adding a chromogenic substrate, the presence of reactive antigen specific T cells was revealed as a spot on the well. Automated spot counting was performed using a 166 CTL ELISPOT system (CTL-ImmunoSpot® S5 Versa Analyzer, USA). The results were recorded based on the definition of positive and negative reactions given in the instructions from the manufacturer. Responses were scored positive if the test wells contained a mean of at least six spot-forming cells more than the mean of the negative control wells, and if this number was at least twice the mean of the negative control wells.

### Statistical Analysis

Data analysis was performed using SPSS, Version 17 (SPSS Inc). Categorical data were compared by Pearson’s Chi-squared or Fisher’s exact test. Odds ratio and multiple logistic regression analysis were used to calculate crude and adjusted risk, and model building was performed backward using the chance criteria for variable selection. Covariates that were significant in bivariate analyses were included in the preliminary model. In addition, other covariates that were considered biologically important were forced into the model irrespective of statistical significance. All p-values reported were calculated two-tailed with statistical significance set to *p*≤0.05.

## Results

### Study Population

**Figure 1:**
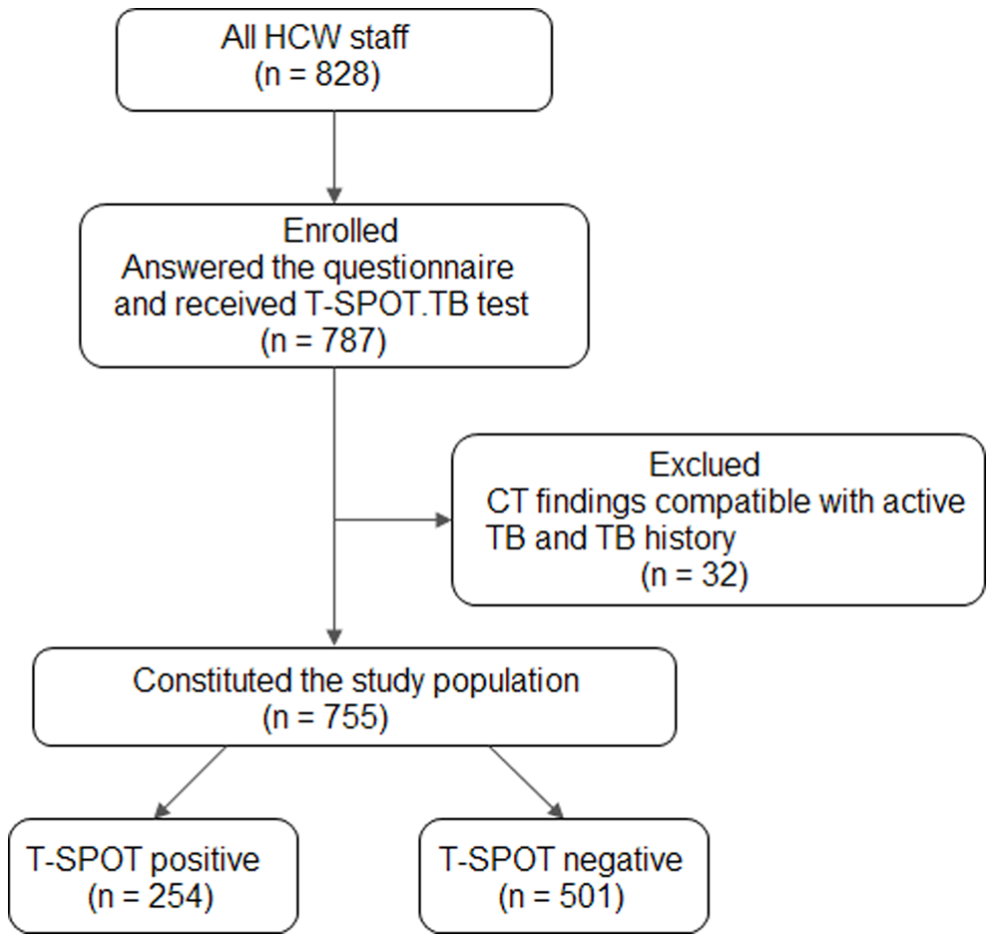
Study flow diagram. Of 828 HCWs, 787 HCWs answered the questionnaire and agreed to be tested for LTBI. 29 individuals with CT findings compatible with active TB and 3 individuals with TB history were excluded. 755 participants were eligible to be included in the final analyses. HCWs: health care workers; LTBI: latent tuberculosis infection; CT: computed tomography; TB: tuberculosis.

Figure 1 shows the flow diagram of the study recruitment. 787 individuals in total were enrolled in the present study. 29 individuals (3.7%) with CT findings compatible with active TB and three individuals with TB history (0.4%) were excluded from the analysis. Finally, 755 participants (95.9%) constituted the study population (Figure 1). The demographic features of the study population are shown in Table 1. The mean age was 39 years (range, 19–66 years), 525 (69.5%) were women, and the mean working duration was 16 years (range, 1–43 years). All of the respondents had received BCG vaccination. More details of the study population are available in Dataset S1 (XLS).

**Table 1:** Description of study population.

Variable		n	(%)
Age	<30	171	(22.6%)
	30–39	201	(26.6%)
	40–49	269	(35.6%)
	≥50	114	(15.1%)
Gender	Male	230	(30.5%)
	Female	525	(69.5%)
Education	University and higher	312	(41.3%)
	Diploma and lower	443	(58.7%)
Working years	≤2	74	(9.8%)
	3–5	101	(13.4%)
	6–10	98	(13.0%)
	11–20	184	(24.4%)
	>20	298	(39.5%)
Job	Administration staff	135	(17.9%)
	Technician staff	127	(16.8%)
	Laboratory staff	76	(10.1%)
	Doctors	125	(16.6%)
	Nurses	292	(38.7%)
Workplace	Low risk workplaces	373	(49.4%)
	High risk workplaces	382	(50.6%)
History of household	No	723	(95.8%)
TB contact	Yes	32	(4.2%)
Laboratory staff	Non-TB lab	53	(7.0%)
	TB lab	23	(3.0%)

TB: tuberculosis.

### LTBI Prevalence

In this study, the prevalence rate of LTBI among all participants was 33.6% (95% CI: 30.2%; 37.0%). The LTBI rate in the chest hospital (33.8%) appeared higher than in the TB research institute (31.8%), however, this was not statistically significant (*p* = 0.743) (data not shown). Regarding the prevalence of LTBI in different workplaces, we found the proportion of LTBI was significantly higher among the high risk workplaces (37.4%) compared to the low risk workplaces (29.8%) (*p* = 0.026).

The highest prevalence was found among laboratory staff (43.4%), followed by technician staff (39.4%), doctors (34.4%) and nurses (32.2%), and the lowest was observed in administrative staff (25.2%). The LTBI rate among TB laboratory staff (39.1%) was lower than non-TB laboratory staff (45.3%), with no statistically significant difference between the groups (*p* = 0.619).

**Figure 2:**
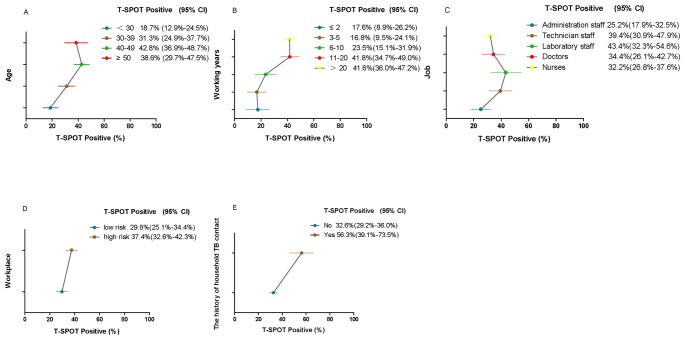
Prevalence of LTBI in HCWs, stratified by age, working years, job, workplace, and the history of household TB contact. The circles and the lines represent the T-SPOT positive rates and 95% CIs, respectively. In univariable analysis, age (A), working years (B), job (C), workplace (D) and the history of household TB contact (E) were significantly associated with LTBI. LTBI: latent tuberculosis infection; HCWs: health care workers; TB: tuberculosis.

The duration of employment had a significant impact on the prevalence of LTBI. Positive T-SPOT.TB test results accounted for 17.6%, 16.8%, 23.5%, 41.8% and 41.6% in groups of ≤2, 3–5, 6–10, 11–20, and >20 working years respectively (*p* = 0.000). The proportion of LTBI was significantly higher among those with the history of household TB contact (56.3%) compared to those without the history of household TB contact (32.6%) (*p* = 0.008). (Figure 2).

### Factors Associated with LTBI

In univariable analysis, non-occupational factors found to be significantly associated with LTBI were aged among 30 to 39 years old [1.98 (95% CI: 1.22; 3.23)], 40 to 49 years old [3.24 (95% CI: 2.06; 5.11)] and 50 years and above [2.73 (95% CI: 1.59; 4.68)]. Another significant predictor variable was the history of household TB contact [2.65 (95% CI: 1.30; 5.43)]. No statistically significant association was observed for gender and education level. Among occupational factors analyzed, the risk factors found to be significantly associated with higher prevalence of LTBI were working duration 11 to 20 years [3.38 (95% CI: 1.73; 6.58)], and 20 years above [3.34 (95% CI: 1.76; 6.35)]. We also identified risk factors for LTBI among job categories, including administration staff, laboratory staff, technician staff, doctors and nurses. Laboratory staff [2.28 (95% CI: 1.25; 4.14)] and technician staff [1.93 (95% CI: 1.14; 3.27)] were associated with increased risk of LTBI while doctors and nurses were not associated with increased risk of LTBI. Using the subgroup with non-TB laboratory staff as comparison group, TB laboratory staff was not significant risk factor for LTBI. In addition, working in high risk workplaces showed significant association with LTBI.

In multivariate analysis, only three factors were significantly associated with LTBI: job categories (Laboratory staff [2.76 (95% CI: 1.36; 5.60)], technician staff [2.02 (95% CI: 1.12; 3.64)]); as a HCW for 11 to 20 years [3.57 (95% CI: 1.46; 8.71)], and 20 years above [3.41 (95% CI: 1.28; 9.11)]; and the history of household TB contact [2.47 (95% CI: 1.15; 5.33)]. (Table 2).

**Table 2:** Association between risk factors and positive T-SPOT.TB by means of univariate and multivariate analysis.

		T-SPOT.TB positive					
Risk factors		results		Uni-variate		Multi-variate	
		n	(%)	OR	(95% CI)	OR*	(95% CI)
Age	<30	32/171	(18.7)	1.00	(reference)	1.00	(reference)
	30–39	63/201	(31.3)	**1.98**	(1.22–3.23)	0.96	(0.48–1.93)
	40–49	115/269	(42.8)	**3.24**	(2.06–5.11)	1.17	(0.51–2.66)
	≥50	44/114	(38.6)	**2.73**	(1.59–4.68)	0.97	(0.36–2.58)
Gender	Male	81/230	(35.2)	1.00	(reference)	1.00	(reference)
	Female	173/525	(33.0)	0.90	(0.65–1.25)	0.81	(0.52–1.25)
Education	University and higher	97/312	(31.1)	1.00	(reference)	1.00	(reference)
	Diploma and lower	157/443	(35.4)	1.22	(0.89–1.66)	1.20	(0.81–1.80)
Working years	≤2	13/74	(17.6)	1.00	(reference)	1.00	(reference)
	3–5	17/101	(16.8)	0.95	(0.43–2.10)	0.96	(0.43–2.17)
	6–10	23/98	(23.5)	1.44	(0.67–3.08)	1.45	(0.64–3.30)
	11–20	77/184	(41.8)	**3.38**	(1.73–6.58)	**3.57**	(1.46–8.71)
	>20	124/298	(41.6)	**3.34**	(1.76–6.35)	**3.41**	(1.28–9.11)
Job	Administration staff	34/135	(25.2)	1.00	(reference)	1.00	(reference)
	Technician staff	50/127	(39.4)	**1.93**	(1.14–3.27)	**2.02**	(1.12–3.64)
	Laboratory staff	33/76	(43.4)	**2.28**	(1.25–4.14)	**2.76**	(1.36–5.60)
	Doctors	43/125	(34.4)	1.56	(0.91–2.66)	1.90	(0.97–3.71)
	Nurses	94/292	(32.2)	1.41	(0.89–2.23)	1.55	(0.84–2.89)
Workplace	Low risk workplaces	111/373	(29.8)	1.00	(reference)	1.00	(reference)
	High risk workplaces	143/382	(37.4)	**1.41**	(1.04–1.91)	1.25	(0.87–1.80)
The history of	No	236/723	(32.6)	1.00	(reference)	1.00	(reference)
household TB contact	Yes	18/32	(56.3)	**2.65**	(1.30–5.43)	**2.47**	(1.15–5.33)
Laboratory staff	Non-TB lab	24/53	(45.3)	1.00	(reference)		
	TB lab	9/23	(39.1)	0.78	(0.29–2.11)		

OR: odds ratio; CI: confidence interval; TB: tuberculosis.

* From a multivariate logistic regression model with age, gender, education, working years, job, workplace, the history of household TB contact.

## Discussion

This is the first study, to our knowledge, to evaluate the prevalence of LTBI in HCWs in China using IGRA. This prevalence of LTBI (33.6%) was lower than the finding assumed in the past with the TST (55.6%) in China [9]. TB disease rate in our study (3.7%) was higher than this Chinese survey (1.5%). This may be explained by the fact that this survey used chest X-ray, which has lower sensitivity than chest CT for detecting TB [18]. Our data were also lower compared to other studies in low and middle income countries with a pooled prevalence estimate of 54% [8]. These reports were almost based on the use of the TST (Only one study used an IGRA for estimation of prevalence among HCWs), so far, several systematic findings of LTBI in HCWs using TST and IGRA have shown a high proportion of TST-positive/IGRA-negative results, which was most likely explained by BCG vaccination [19], [20]. Therefore, the discrepancy between our finding and those with the use of the TST is most likely due to the BCG-vaccination. The main limitation of our study is the cross-sectional study design. Some of the participants have a history of working in other departments prior to their current workplace, and we could not determine the timing of infection.

The overall prevalence of IGRA positivity (33.6%) among HCWs in our study was lower compared to previous estimates from other high-incidence TB countries which used IGRA for diagnosing LTBI. Pai et al. [16] investigated 726 HCWs from an Indian rural medical school who were screened for TB infection using the IFN-γ assay and TST. The prevalence of IFN-γ assay positivity was 40.0%. Drobniewski et al. [21] assessed the prevalence and risk factors for LTBI among students, primary care health providers, and TB hospital health providers in Russian Federation with QuantiFERON–TB Gold assay. LTBI was seen in 40.8% of all health care staff. A report from Viet Nam [14] observed 300 HCWs from two hospitals (a TB hospital and a non-TB hospital) received QuantiFERON-TB Gold, followed by one- and two-step TST. The LTBI rate estimated by IGRA was 47.3%. The lower prevalence in our study could be attributed to the inclusion of more administrative workers who are at lower risk of exposure to TB in the work-place than these studies from Russia and Viet Nam. No administrative workers were recruited in the Indian research. However, the prevalence might be an underestimate because they recruited few senior physicians who were older and, presumably, more frequently exposed to MTB [16].

Compared to studies which used IGRA for diagnosing LTBI in countries with an intermediate and low incidence of TB, the LTBI rate of our finding was higher. Hirama and colleagues [22] demonstrated that the proportion of positive IGRA among HCWs in the Saitama Medical University Hospital in Japan was 6.64%. The prevalence among 2028 HCWs in 14 different kinds of hospitals in Germany was 9.9%. The highest prevalence of LTBI in this German study was among the administration staff (17.4%) followed by ancillary nursing staff (16.7%) [19]. Similar finding was reported by a Malaysian research whereby the prevalence of LTBI among the administration staff was 10.7%, second only to medical ward (13.7%) and emergency department workers (11.6%) [5]. In contrast to these results, the prevalence of LTBI among the administration staff in our data was the lowest rate. Whether there is increased risk of LTBI among HCWs in non-direct contact with TB patients, further researches need to be conducted. In addition, we focused on HCWs in a TB specialist hospital, while none of the above studies (Japan, Germany and Malaysia) were conducted in a TB specialist hospital. Thus, HCWs in TB specialist hospitals and high TB incidence countries are likely to experience heavy TB exposure, and this may explain a higher LTBI prevalence in our study.

Another goal of this study was to assess risk factors for LTBI among HCWs. In univariable analysis, our study found that LTBI was associated with age, working duration, job category, workplace and the history of household TB contact. Our results are consistent with other reports that increasing age and more years of employment are associated with higher prevalence of LTBI [23], [24]. In multivariable analysis, age was no longer significantly associated with LTBI. This is probably because the risk of infection depends primarily on the duration of exposure to TB [7].

Years in health care reflect cumulative exposure to MTB, and variability of risk across job categories may reflect variations in exposure frequency and intensity [16]. A recently published result from Germany [25] showed job category was a significant predictor for LTBI, but the detailed categories were different from our data. The German report indicated that the occupational LTBI risk was physician or nurse. In our study, multivariable analysis showed laboratory staff and technician staff were associated with increased risk of LTBI while doctor and nurse were not the risk factors. Direct comparison is difficult, because job categories of the German report are nurse, physician and other professions, while our job categories are administration staff, laboratory staff, technician staff, doctor and nurse, which may affect the analysis of predictors. A Polish study found a higher risk of acquiring LTBI was associated with TB lab workers (50%) and TB ward staff (34%), while the incidence of LTBI was lowest in administration staff [26]. In line with the literature, in our study, the highest prevalence of LTBI was in lab workers (43.4%) and the lowest prevalence of LTBI was in administration staff (24.8%), but we could not distinguish between TB lab staff and non-TB lab staff. Between groups of workers, there are major differences. Nevertheless, laboratory personnel should use universal precautions for handling specimens, particularly for MTB. In addition, our next research will be concentrated on exploring the differences between job categories, and on identifying potential risk groups.

In multivariable analysis, workplace was not significantly associated with LTBI. However, the proportion of LTBI was higher among the high risk workplaces (37.4%) compared to the low risk workplaces (29.8%), and univariable analysis showed that working in high risk workplaces was associated with higher prevalence of LTBI. Meanwhile, some previous studies have shown an occupational risk factor was linked to the workplace [27], [28]. Therefore, workplace was considered biologically important. Similar to other reports [5], [29], in our study, the history of household TB contact was significantly associated with LTBI in multivariable analysis. Living in the same house with patients who had TB disease is a known risk factor as these individuals share the same air space and are in close contact with each other. In addition, because all subjects in our study were BCG vaccinated, we couldn’t analyse the association between BCG vaccination and positive T-SPOT.TB. Nevertheless, BCG status was not a predictor for LTBI according to several previously reports [23], [25], [30], [31].

In conclusion, we found a high prevalence of LTBI among HCWs in a hospital for pulmonary diseases and a TB research institute in China using the IGRA. Multivariable analysis showed working duration, job category, and the history of household TB contact were significantly associated with LTBI. These observations suggest that adequate infection control measures and staff health monitoring should be implemented to protect HCWs. With the recent emergence of extensively drug-resistant tuberculosis (XDR-TB), the need to implement infection-control programs has been reemphasized by global agencies such as the WHO and the Stop TB Partnership [32]. Because of a high TB prevalence, China should pay more attention to tackle the problem of nosocomial TB and reduce nosocomial transmission. Moreover, further researches need to be conducted to analyze the risk of LTBI among HCWs in non-direct contact with TB patients and explore the differences between job categories.

## Supporting Information

Dataset S1 (XLS)
